# Acknowledgment to Reviewers of *Epigenomes* in 2020

**DOI:** 10.3390/epigenomes5010004

**Published:** 2021-01-28

**Authors:** Epigenomes Editorial Office

**Affiliations:** MDPI AG, St. Alban-Anlage 66, 4052 Basel, Switzerland

Peer review is the driving force of journal development, and reviewers are gatekeepers who ensure that *Epigenomes* maintains its standards for the high quality of its published papers. Thanks to the cooperation of our reviewers, in 2020, the median time to first decision was 16 days and the median time to publication was 41 days. The editors would like to express their sincere gratitude to the following reviewers for their precious time and dedication, regardless of whether the papers were finally published:
Aguilar-Arnal, LorenaKadalayil, LathaBedford, MarkKaundal, RavinderBerg, Matthew D.Kim, Byung SooBerr, AlexandreKubala, SzymonBlanco, EnriqueLeitão, José M.Castillo-Fernandez, JuanLim, Hee-WoongChan, MichaelMancera-Páez, OscarCiabrelli, FilippoMartino, SabataColacino, Justin A.Maury, StéphaneCorces, Victor G.McKeon, PeterCrisp, PeterMinoda, AkiD’Agostino, Vito GiuseppeMontes Resano, MartaDar, Mohd SaleemMorey, LluisDe La Serna, Ivana L.Muniandy, MaheswaryDing, LinNakabayashi, KazuhikoEggermann, ThomasNowicka, AnnaErokhin, MaksimOlova, NellyFeil, RobertPanda, AmareshGackowski, DanielPouliot, RoxaneGao, XuPrimig, MichaelGazouli, MariaPydi, Sai PrasadGreinert, RüdigerRichtig, GeorgHedrich, ChristianRobinson, Gene E.Heitkam, TonyRoquis, DavidHeller, GerwinRotblat, BarakHwan-young, LeeSancisi, ValentinaIgotti, MariaSharma, SapnaIshibashi, ToyotakaSpeijer, DaveIsles, Anthony R.Strong, Cristina De GuzmanJaguva Vasudevan, Ananda AyyappanTzelepi, VasilikiJayanthi, SankarUlicna, LiviaJayaraman, SundararajanVan Haute, LindseyJeong, Dong-HoonVerde, GaetanoJi, HongVicentini, CaterinaJing, RanXie, WeiJohnson, BenYan, QinJurkowska, Renata ZofiaZaph, Colby

